# Valsartan blocks thrombospondin/transforming growth factor/Smads to inhibit aortic remodeling in diabetic rats

**DOI:** 10.1186/s13000-015-0246-8

**Published:** 2015-04-02

**Authors:** Hui Sun, Yong Zhao, Xiuping Bi, Shaohua Li, Guohai Su, Ya Miao, Xiao Ma, Yun Zhang, Wei Zhang, Ming Zhong

**Affiliations:** The Key Laboratory of Cardiovascular Remodeling and Function Research, Chinese Ministry of Education and Chinese Ministry of Public Health, the Department of Cardiology, Shandong University, Qilu Hospital, No.107, Wen Hua Xi Road, Jinan, Shandong Province 250012 China; Department of Cardiology, Jinan Central Hospital Affiliated to Shandong University, Jinan, 250013 China; Department of Geriatric Cardiology, Provincial Hospital Affiliated to Shandong University, Jinan, 250021 China

**Keywords:** Diabetes, Macrovascular remodeling, Thrombospondin 1, Transforming growth factor β1, Smads, valsartan

## Abstract

**Background:**

Angiotensin II (Ang II) and transforming growth factor β (TGFβ) are closely involved in the pathogenesis of diabetic complications. We aimed to determine whether an aberrant thrombospondin 1 (TSP1)–mediated TGFβ1/Smads signaling pathway specifically affects vascular fibrosis in diabetic rats and whether valsartan, an Ang II subtype 1 receptor blocker, has an anti-fibrotic effect.

**Methods:**

Age-matched male Wistar rats were randomly divided into 3 groups: control (n = 8), diabetes (n = 16) and valsartan (30 mg/kg/day) (n = 16). Type 2 diabetes mellitus (T2DM) was induced by a high-calorie diet and streptozotocin injection. Morphological and biomechanical properties of the thoracic aorta were assessed by echocardiography and cardiac catheterization. Masson staining was used for histological evaluation of extracellular matrix (ECM). The expression of components in the TSP1–mediated TGFβ1/Smads signaling pathway was analyzed by immunohistochemistry and real-time quantitative reverse transcription polymerase chain reaction.

**Results:**

As compared with controls, diabetic aortas showed reduced distensibility and compliance, with excess ECM deposition. Components in the TSP1-mediated TGFβ1/Smads signaling pathway, including TSP1, TGFβ1, TGFβ type II receptor (TβRII), Smad2 and Smad3, were accumulated in vascular smooth muscle cytoplasm of diabetic aortas and their protein and mRNA levels were upregulated. All these abnormalities were attenuated by valsartan.

**Conclusions:**

TSP1-mediated TGFβ1/Smads pathway activation plays an important role in marcovascular remodeling in T2DM in rat. Valsartan can block the pathway and ameliorate vascular fibrosis.

**Virtual slides:**

The virtual slide(s) for this article can be found here: http://www.diagnosticpathology.diagnomx.eu/vs/1053842818141195

## Background

Diabetes is a serious health problem worldwide, and the total number of diabetic patients is projected to increase from 171 million in 2000 to 366 million in 2030 [1]. A growing body of evidence supports diabetes associated with various cardiovascular complications, including macro- and microangiopathies. Hyperglycemia-induced unfavorable remodeling has been reported in the thoracic aorta [2], coronary artery [3], renal vasculature [4] and intestinal arterioles [5] of diabetic animal models. Vascular remodeling, characterized by alterations in the composition and assembly of extracellular matrix (ECM), is involved in accelerated atherosclerosis.

Transforming growth factor β (TGFβ) plays a critical role in modulating the synthesis and degradation of ECM. It is secreted as a latent complex (L-TGFβ), which contains a latency-associated peptide (LAP) and a C-terminal bioactive region. On stimulation with multiple factors by enzymatic cleavage of or physical interaction with LAP, an active form of TGFβ (A-TGFβ) is released from its latent precursor. A-TGFβ exerts its effects on target genes by binding to specific receptors (TβRs) and subsequent phosphorylation of Smads [6,7]. Experimental and clinical studies indicate that hyperglycemia stimulates the production of TGFβ1, thrombospondin 1 (TSP1), and angiotensin II (Ang II) in the diabetic condition [8,9].

TSP1 is a matricellular protein involved in ECM formation. It can activate TGFβ1 endogenously by binding to the LAP and mature domain of TGFβ1 [10]. TSP1-mediated TGFβ1/Smads signaling contributes to target-organ damage in animals with diabetic nephropathy [11] and diabetic cardiomyopathy [12]. In addition, glucose or Ang II alone or in combination upregulates TSP1 and elevates TGFβ1 activity. These effects can be antagonized by Ang II subtype 1 receptor blockers (ARBs), which suggests stimulation of the renin–angiotensin system (RAS) in the development and progression of renal and cardiac fibrosis [13].

However, we lack information on macrovascular lesions provoked by TSP1 in diabetes. Therefore, we hypothesized that hyperglycemia promotes the accumulation of ECM in the thoracic aorta through an Ang II-TSP1-TGFβ1/Smads pathway and examined whether valsartan, an ARB widely used in clinical practice, could reverse such arterial remodeling in rat.

## Methods

### Animal model

Age-matched male Wistar rats (200–240 g, 48–50 days) obtained from Shandong University Laboratories Animal Center (Jinan, China) were randomly divided into 3 groups: control (n = 8), diabetic (n = 16) and valsartan (n = 16). Animals in the control group were fed normal chow (8% fat, 16% protein, 50% carbohydrate, and 22% other ingredients; total calories 14 kJ/g) and the other 2 groups a high-calorie diet (25% fat, 14% protein, 51% carbohydrate, and 10% other ingredients; total calories 20 kJ/g). Four weeks later, venous blood was sampled for measuring fasting plasma glucose (FPG) and fasting insulin (Ins). After another week, streptozotocin (STZ; Sigma, St. Louis, MO; 30 mg/kg, dissolved in ice-cold 10 mM citrate buffer, pH 4.4) was administered intraperitoneally to diabetic and valsartan groups, and an equivalent volume of citrate buffer was administered to the control group. One week after STZ injection, blood samples were collected from the tail vein for measuring FPG and Ins. Diabetes was defined as FPG ≥11.1 mmol/L and insulin sensitivity index [ISI = Ln(FPG × Ins)^-1^] lower than that of controls. Rats in the valsartan group were given valsartan (30 mg/kg) via intragastric administration every day, and those in control and diabetic groups received the same dose of normal saline. Animals were maintained in individual air-filtered metabolic cages with free access to water for 16 weeks. FPG and Ins were measured at the end of the experiment, with ISI calculated. This study conformed to the *Guide for the Care and Use of Laboratory Animals* published by the US National Institutes of Health (NIH Publication No. 85–23, revised 1996). The protocol was granted by the institutional medical ethics review board.

### Echocardiography and cardiac catheterization

At the end of the experiment, rats were anesthetized with chloral hydrate (300 mg/kg, intraperitoneally). Transthoracic echocardiography was performed with use of a SONOS 7500 (Hewlett-Packard, Andover, MA, USA) with a 12 MHz transducer. The inner diameter of the thoracic aorta was measured in systolic and diastolic phases (Ds, Dd) in a long axis view. Subsequently, a catheter (PE-50) was introduced into the aortic arch via the right carotid artery and connected to a pressure transducer for measuring aortic systolic blood pressure (SBP) and diastolic blood pressure (DBP). Aortic distensibility and compliance were determined by calculating distensibility coefficient (DC) and compliance coefficient (CC), respectively, by the following formulas [14,15]:

$$ \mathrm{D}\mathrm{C}=\left(\varDelta \mathrm{A}/\mathrm{A}\right)/\varDelta \mathrm{P}=2\left(\varDelta \mathrm{D}\mathrm{x}\mathrm{D}\mathrm{d}+\varDelta {\mathrm{D}}^2\right)/\left(\varDelta \mathrm{P}\mathrm{x}\mathrm{D}{\mathrm{d}}^2\right). $$

$$ \mathrm{C}\mathrm{C}=\left(\varDelta \mathrm{V}/\varDelta \mathrm{L}\right)/\varDelta \mathrm{P}=\mathrm{A}/\mathrm{P}=\uppi \left(2\varDelta \mathrm{DxDd}+\varDelta {\mathrm{D}}^2\right)/4\varDelta \mathrm{P}. $$

$$ \varDelta \mathrm{P}=\mathrm{S}\mathrm{B}\mathrm{P}\hbox{-} \mathrm{D}\mathrm{B}\mathrm{P}.\ \varDelta \mathrm{D}=\mathrm{D}\mathrm{d}\hbox{-} \mathrm{D}\mathrm{s}. $$

### Tissue preparation

Animals were killed by an overdose of chloral hydrate. The thoracic aorta was excised immediately and dropped into an ice-cold NaCl 0.9% buffer. Tissues (5 × 5 × 5 mm^3^ aortic wall) for immunohistochemistry were fixed in 10% formaldehyde and paraffin embedded. The remaining aorta was cut into small tissue blocks and stored in foil packets at −80 °C for the following experiments.

### Histological evaluation of extracellular matrix (ECM)

Sections 4 μm thick were deparaffined and stained with Masson’s trichrome for ECM. Ten successive microscopy fields were examined with use of the JD801 Imaging Analysis System (Jiangsu JEDA Science-Technology Development Co.). The content of aortic ECM was semi-quantified as the proportion of area occupied by Masson’s staining to total area.

### Immunohistochemistry

Immunohistochemistry involved a microwave-based antigen-retrieval technique. After the removal of paraffin, endogenous peroxidase was neutralized with H_2_O_2_ (0.3% vol/vol) for 10 min. Sections were placed in phosphate-buffered saline (PBS) for 15 min and protein-blocking solution (Immunotech, Cedex, France) for another 30 min, incubated with primary antibodies overnight at 4°C, then with secondary antibodies for 1 hour at room temperature, and finally horseradish peroxidase–conjugated streptavidin (Dako; diluted 1:500) for visualization. The expression of TSP1, L-TGFβ1, A-TGFβ1, TβRII and p-Smad2/3 was evaluated by use of the JD801 imaging analysis system. The percentage positive staining in the vascular wall was semi-quantified under a microscope.

### Real time quantitative reverse transcription polymerase chain reaction (RT-PCR)

Total RNA was extracted from aortic tissues by use of Trizol reagent and treated with DNase to avoid DNA contamination. After quantification at the extinction coefficient of 260 nm, total RNA was reverse-transcribed into cDNA following the manufacturer’s instructions (TakaRa, Dalian, China), and real-time PCR involved an ABI Prism 7000 sequence detector system with the SYBR Green Reaction Kit. Primers are in Table 1. Amplification products were analyzed by a melting curve, which confirmed a single PCR product in all reactions. The expression of specific genes was normalized to that of β-actin as the housekeeping gene.

**Table 1:** **cDNA Primer sequences for real-time RT-PCR**

**Signaling components**	**Primers**
TSP 1	Forward: 5’-GGAAGAGCATCACGCTGTTTG-3’
	Reverse: 5’-GCGCTCTCCATCTTGTCACA-3’
TGFβ1	Forward: 5’ TTGCCCTCT ACAACCAACACAA-3’
	Reverse: 5’-GGCTTGCGACCCACGTAGTA- 3’
TβRII	Forward: 5’ TCA CCT ACC ACG GCT TCA CTC T 3’
	Reverse: 5’ CGC CCT TTT CTT TTC CTT CA 3’
Smad2	Forward: 5’- TGT GCA GAG CCC CAA CTG TA -3’
	Reverse: 5’- TGG TGG GAT TTT GCA CAC TGT -3’
Smad3	Forward: 5’- CAA CCC CTC AGG TTC TCTGAA G -3’
	Reverse: 5’- GCA GTC CAC AGA CCA TGT CAA -3’
β-actin	Forward: 5’- TTC AAC ACC CCA GCC ATG T -3’
	Reverse: 5’- GTG GTA CGA CCA GAG GCA TAC A -3’

*Abbreviations*: *TGFβ1* transforming growth factor β1, *TβRII* TGF β type II receptor, *TSP1* thrombospondin 1.

### Statistical analysis

Data are expressed as mean ± SD. Statistical analysis involved use of SPSS 11.0 (SPSS, Chicago, IL), with unpaired Student *t* test for comparisons between 2 groups and ANOVA followed by Scheffe’s procedure for 3 groups. *P* < 0.05 was considered statistically significant.

## Results

### Characteristics of experimental animals

**Figure 1:**
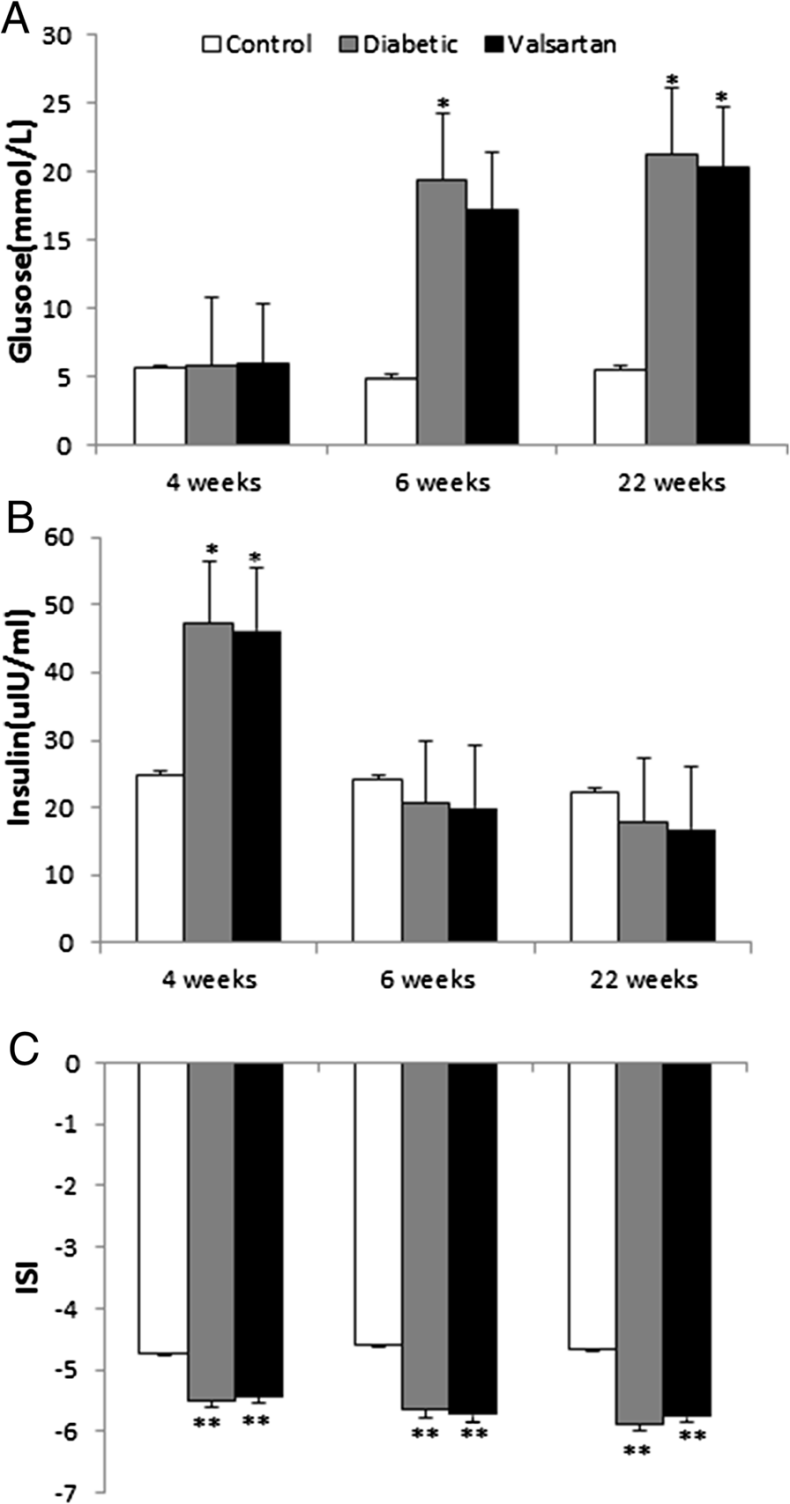
**Biochemical characteristics of thoracic aortas.** Measurements of fasting plasma glucose **(A)** and fasting insulin **(B)** before STZ injection (4 week), 1 week after STZ injection (6 week) and the end of the experiment (22 week), with ISI **(C)** calculated. *P < 0.05 and **P < 0.01, vs controls. Abbreviations: ISI, insulin sensitivity index; STZ, streptozotocin.

During the experiment, 3 rats died in the diabetic group and 2 in the valsartan group. These deaths were attributable to ketoacidosis, infections or other complications induced by hyperglycemia. The FPG and ISI of 3 rats treated with a high-calorie diet and STZ did not meet the definition of diabetes. Finally, 8 rats were included in control group, 11 in diabetic group, and 13 in valsartan group. Biochemical characteristics, including FPG, Ins, and ISI, were similar between diabetic and valsartan groups across the experiment. However, as compared with controls, diabetic and valsartan groups showed significantly elevated Ins before STZ injection (*P* < 0.05), higher FPG one week after STZ injection (*P* < 0.01), and consistently lower ISI (*P* < 0.01; Figure 1).

### Morphological and biomechanical properties of thoracic aortas

Compared with controls, diabetic aortas were enlarged in systolic and diastolic diameters (*P* < 0.01) but reduced in distensibility and compliance (*P* < 0.05), which suggested macrovascular remodeling (Table 2). As compared with diabetic aortas, valsartan aortas showed increased distensibility and compliance (*P* < 0.05) and reduced systolic and diastolic diameters, but not significantly, which indicated some improvement in remodeling with valsartan.

**Table 2:** **Morphological and biomechanical properties of rat thoracic aorta with diabetes or valsartan treatment**

**Groups**	**Dd (mm)**	**Ds (mm)**	**Ps (mmHg)**	**Pd (mmHg)**	**DC (1/kPa)**	**CC (mm** ^**2**^ **/kPa)**
Control	1.58 ± 0.14	1.86 ± 0.22	82.67 ± 19.472	64.00 ± 20.993	0.18 ± 0.10	0.48 ± 0.21
Diabetic	1.86 ± 0.18**	2.18 ± 0.18**	97.37 ± 15.011*	71.50 ± 13.135	0.11 ± 0.05*	0.33 ± 0.17*
Valsartan	1.75 ± 0.18*	2.08 ± 0.17*	71.22 ± 19.642^△^	54.89 ± 19.161^△^	0.24 ± 0.14^△^	0.53 ± 0.31^△^

*Abbreviations*: *CC* compliance coefficient, *DC* distensibility coefficient, *Dd* diastolic diameter, *Ds* systolic diameter.

* *P* < 0.05 vs control; ** *P* < 0.01 vs control; ^∆^ *P* < 0.05 vs diabetic.

### Fibrosis in thoracic aortas

**Figure 2:**
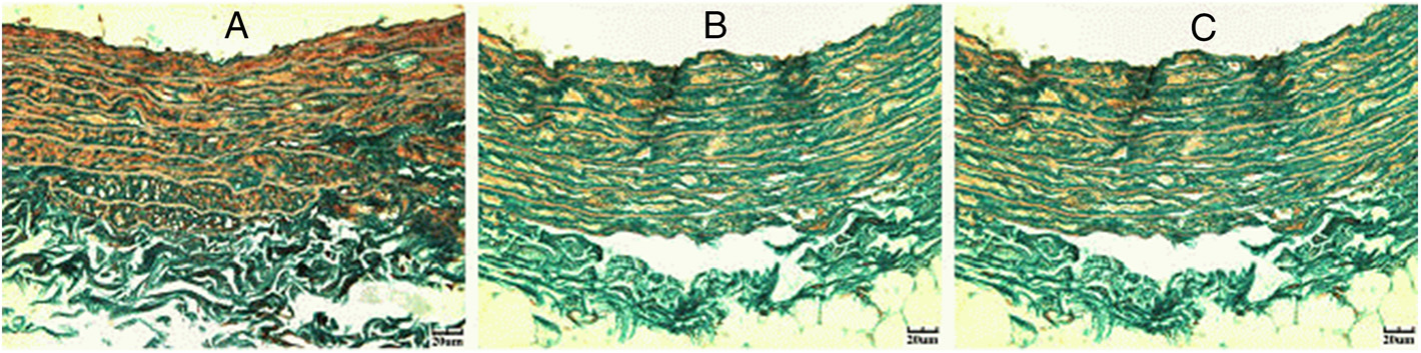
**Masson’s staining of thoracic aortas.** Control **(A)**, diabetic **(B)** and valsartan **(C)** aortas showing extracellular matrix (green).

Masson staining demonstrated well-arranged aortic fibrous tissue in control rats (Figure 2A). The diabetic group showed disarranged fibers (Figure 2B). However, histological manifestations were attenuated in the valsartan group as compared with the diabetic group (Figure 2C).

The content of ECM in the thoracic aorta was higher in diabetic rats than controls (25.73 ± 4.85% vs. 17.12 ± 4.65%; *P* < 0.01). As compared with diabetic aortas, valsartan aortas showed reduced ECM content (20.81 ± 5.41% vs. 25.73 ± 4.85%, *P* < 0.05).

### Protein content of components in the TSP1-mediated TGF β1/Smads pathway

**Figure 3:**
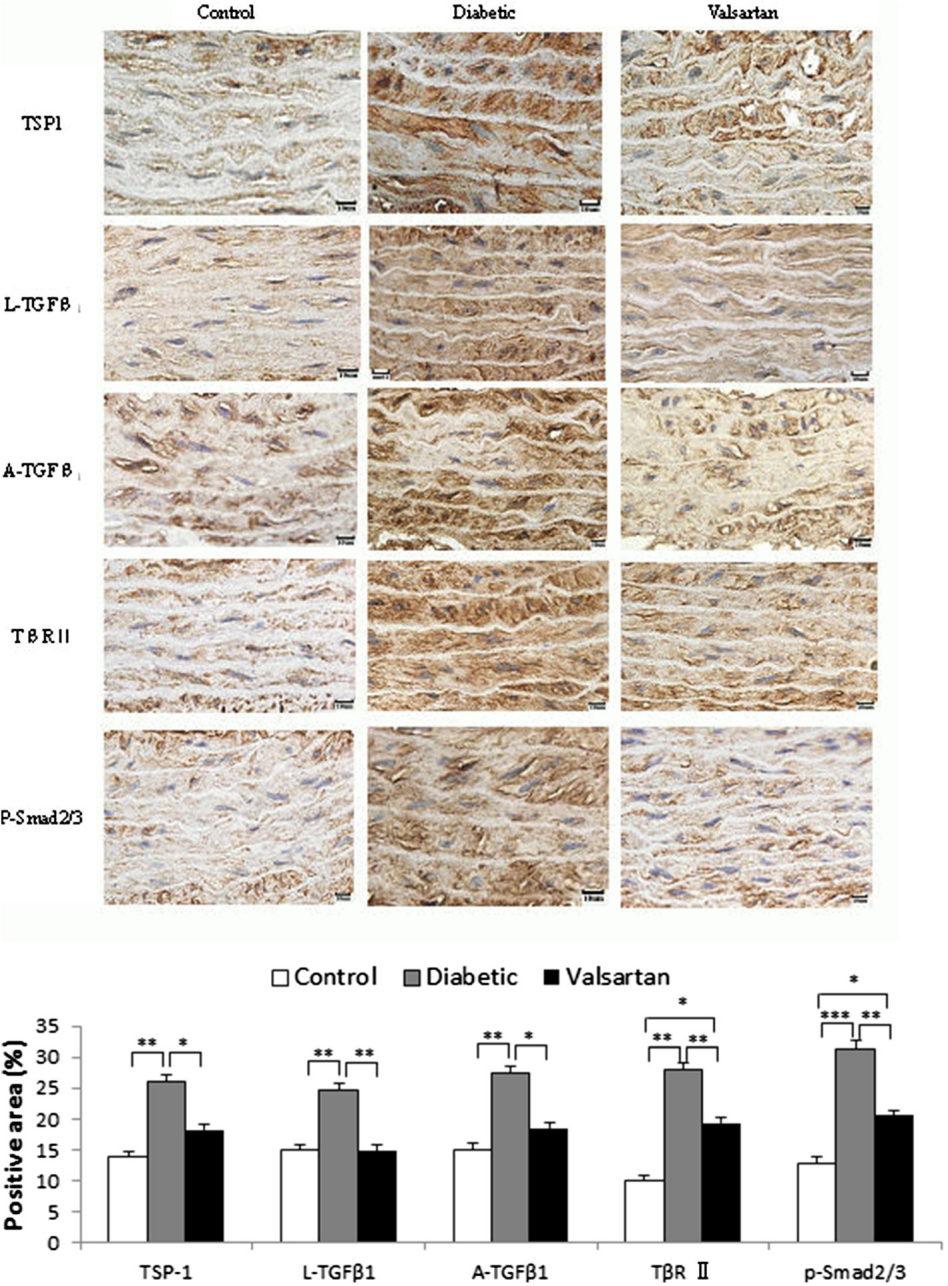
**Immunohistochemistry of protein content of components in the TSP1-mediated TGFβ1/Smads signaling pathway in aortas.** Staining for TSP1, A-TGFβ1, L-TGFβ1, TβRII, and p-Smad2/3 in aortic medial layer of control, diabetic and valsartan aortas and quantification (bottom). *P < 0.05, **P < 0.01, ***P < 0.001. Abbreviations: A-TGFβ1, active transforming growth factor β1; L-TGFβ1, latent transforming growth factor β1; p-Smad2/3, phosphorylated Smad2/3; TβRII, TGFβ type II receptor; TSP1, thrombospondin 1.

On immunohistochemistry (Figure 3), staining for TSP1, L-TGFβ1 and A-TGFβ1, TβRII, and phosphorylated Smad 2/3 (p-Smad2/3) in vascular smooth muscle cytoplasm was high in diabetic aortas, moderate in valsartan aortas and low in controls.

### Transcription of components in TSP1-mediated TGF β1/Smads pathway

**Figure 4:**
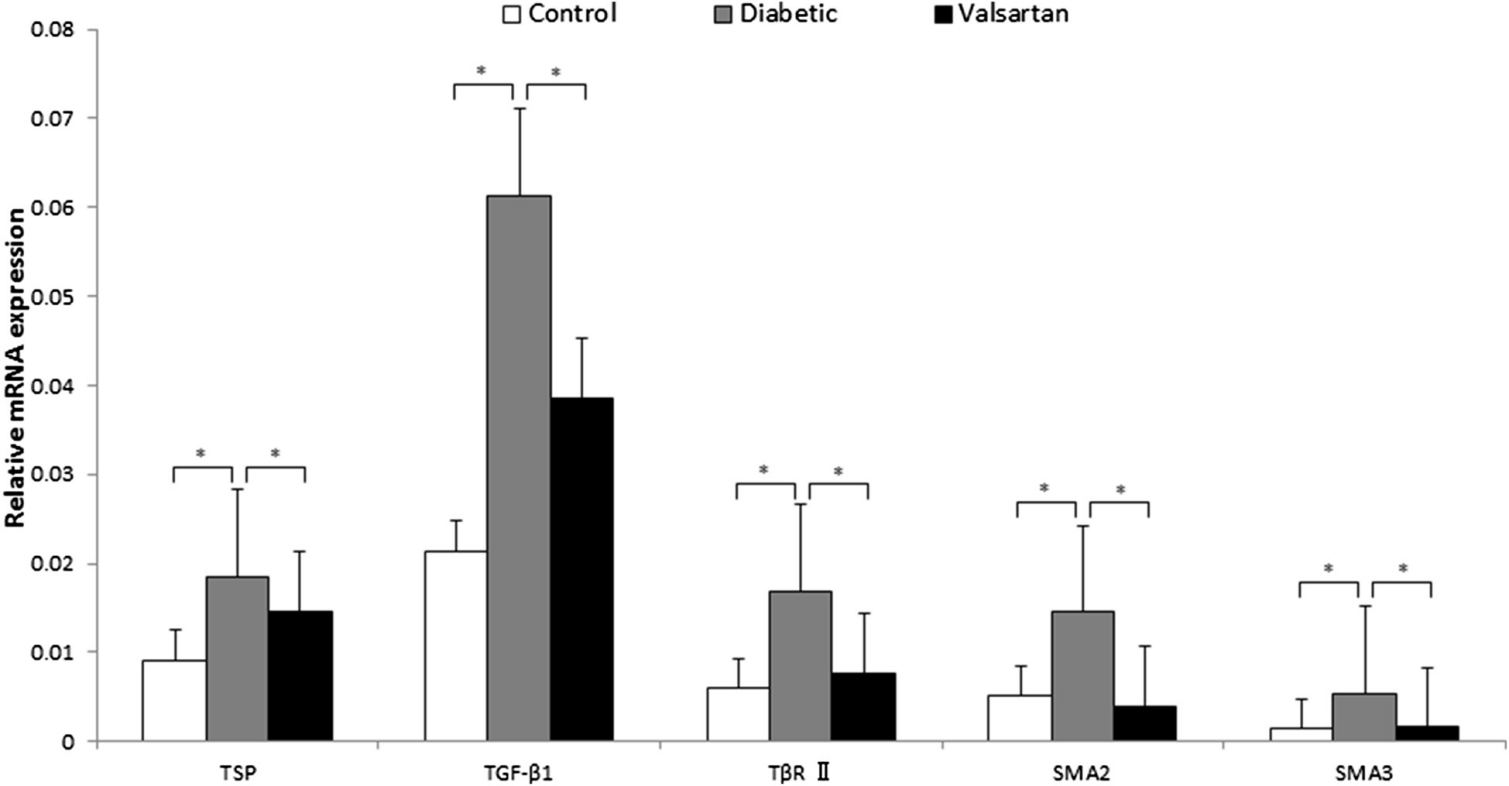
**RT-PCR analysis of mRNA level of components in the TSP1-mediated TGF β1/Smads signaling pathway in aortas.** *P < 0.05. Abbreviations: TGFβ1, transforming growth factor β1; TβRII, TGFβ type II receptor; TSP1, thrombospondin 1.

Real-time quantitative RT-PCR (Figure 4) demonstrated greatly upregulated mRNA levels of TSP1, TGFβ1, TβRII, Smad2 and Smad3 in diabetic than control aortas, with levels in valsartan aortas not significantly different from those in controls.

## Discussion

We examined whether hyperglycemia in diabetic rats promotes the accumulation of ECM in the thoracic aorta through an Ang II-TSP1-TGFβ1/Smads pathway and whether valsartan, an ARB widely used in clinical practice, could reverse such arterial remodeling. Diabetic aortas showed reduced distensibility and compliance, with excess ECM deposition, as compared with controls. Components in the TSP1-mediated TGFβ1/Smads signaling pathway, including TSP1, TGFβ1, TGFβ type II receptor (TβRII), Smad2 and Smad3, were accumulated in diabetic vascular smooth muscle cytoplasm, and their protein and mRNA levels were upregulated. All these abnormalities were attenuated by valsartan. Thus, activation of a TSP1-mediated TGFβ1/Smads pathway plays an important role in marcovascular remodeling in T2DM, and valsartan may hold promise for blocking the pathway and ameliorating vascular fibrosis in diabetes.

Vascular complications of T2DM, including cardiovascular diseases, retinopathy and nephropathy, impose a substantial socioeconomic burden on public health. Approximately 50% of patients with T2DM die prematurely of a cardiovascular cause and 10% die of renal failure [16]. Abnormal arterial remodeling, paralleled by accelerated atherosclerosis, is responsible for the elevated incidence of ischemic complications in diabetes. This process extends to blood vessels of various caliber and leads to an excessive accumulation of ECM. At the macrovascular level, these alterations bring about narrowed lumen, increased stiffness and decreased vasomotion [17]. In the present and previous studies [2], we used a high-calorie diet and low-dose STZ injection to establish an animal T2DM model with specific metabolic characteristics and demonstrated structural and functional remodeling in the thoracic aorta.

The molecular mechanisms of arterial remodeling are not fully elucidated. Multifunctional cytokines seem to play a crucial role. TSP1-dependent TGFβ activation is involved in the development of cardiac fibrosis in rats with diabetes and elevated Ang II level [18]. Our current study showed that TSP1-mediated TGFβ1/Smads signaling is intensively involved in macrovascular fibrosis induced by hyperglycemia. In parallel to upregulated TSP1 mRNA in diabetic aortas, that of the factors A-TGFβ, TβRII, and p-Smad2/3 was upregulated, as was cellular staining, which indicates TGFβ1 signaling activity. TSP1 is an extracellular calcium-binding multifunctional protein first discovered in activated platelets. It is also secreted by endothelial cells and smooth muscle cells. TSP1 triggers the activation but not expression of TGFβ1 by interacting with the LAP of latent TGFβ1 [19]. To initiate its cellular action, TGFβ1 binds to TβRII and TβRI in sequence. After activation, TβRI recruits and phosphorylates the ligand-specific receptor-activated Smads (R-Smads), Smad2 and Smad3, which then form heterometric complexes with a co-Smad, Smad4, for subsequent nuclear signaling. Smad7 is an inhibitory Smad (I-Smad) and inactivates transcription by binding with R-Smads or a co-Smad [20,21]. TGFβ1/Smads signaling modulates ECM by stimulating fibrillar collagen genes and inhibiting matrix metalloproteinase genes [6]. Consistent with findings from rats with diabetic cardiomyopathy [12,18], we observed a significant increase in ECM content with activation of TGFβ1/Smads signaling.

Under high glucose, Ang II production is elevated, with disproportionate matrix deposition [22], which is related to a mechanism dependent on protein kinase C (PKC) [8]. Although we did not determine Ang II level, the increased TSP1-mediated TGFβ1/Smads signaling in diabetic aortas was inhibited by an ARB, valsartan, and the pathological features and biomechanical dysfunction of the diabetic thoracic aorta were substantially improved.

These results suggest an important role of RAS activation in diabetic fibrosis. Early experiments revealed that glucose itself stimulates enhanced TSP1 transcription in the aorta and carotid arteries [23], whereas in mesangial cells, glucose stimulates TSP1 expression and TGFβ activity through nuclear protein USF2 via PKC, p38 mitogen-activated protein kinase (p38 MAPK) and extracellular signal-regulated kinase (ERK) pathways [24]. In a hyperglycemic environment, Ang II stimulates TSP1 upregulation and promotes subsequent activation of TGFβ1. This process is facilitated by the canonical Ang II subtype 1 receptor (AT1R) through p38 MAPK and c-Jun NH2-terminal kinase (c-JNK) but not ERK1/2 [25]. Evidence from our current study and other reports [13,18] suggests that the synergistic effects of glucose and Ang II contribute to increased TSP1 expression and consequent TGFβ1 activation.

The findings in this study that unfavorable morphological and functional alterations in diabetic aortas may be partially reversed by inhibiting the detrimental effects of Ang II are important for clinical practice. It provides new insight into the mechanisms accounting for the vascular benefits of interventions that block RAS overactivation in diabetes. Recent clinical trials demonstrated that stringent control of glycemia decreased the rate of microvascular outcomes [26] but did not reduce major cardiovascular events as compared with standard therapy in high-risk patients with T2DM [27]. In addition, tight control of systolic blood pressure was not associated with improved cardiovascular outcome as compared with usual control treatment [28,29]. However, treatment with an RAS antagonist-based regimen, including an Ang II converting enzyme (ACE) inhibitor or ARB, prevented more cardiovascular events than did other regimens in diabetic patients with or without hypertension [30,31]. Although numerous therapeutic strategies being developed target the TGFβ1/Smads signaling pathway for treating fibrosis, only a few studies have been performed in humans [32,33]. Given the concern about unpredictable side effects of novel therapies, a practical approach for TGFβ1 antagonism is to extend the usefulness of available pharmaceuticals. Tranilast, a membrane-stabilizing agent of mast cells used for treating bronchial asthma, suppresses collagen synthesis in early and advanced diabetic nephropathy by interfering with the actions of TGFβ1 [34,35]. Similarly, as a type of competent antihypertensive agents with favorable tolerability and safety, RAS inhibitors are promising for combating diabetic fibrosis.

## Conclusion

TSP1-mediated TGFβ1/Smads signaling is activated and contributes to the redundant accumulation of ECM induced by hyperglycemia in the rat diabetic thoracic aorta. Blocking the RAS inhibits the expression of signaling components and ameliorates the morphological and biomechanical features of large arteries with diabetes, which suggests an involvement of Ang II. Targeting the Ang II-TSP1-TGFβ1/Smads signaling pathway is a feasible therapeutic option to correct the aberrant macrovascular remodeling in diabetes.
